# Alterations in the microstructure of white matter in children and adolescents with Tourette syndrome measured using tract-based spatial statistics and probabilistic tractography

**DOI:** 10.1016/j.cortex.2018.04.004

**Published:** 2018-07

**Authors:** Hilmar P. Sigurdsson, Sophia E. Pépés, Georgina M. Jackson, Amelia Draper, Paul S. Morgan, Stephen R. Jackson

**Affiliations:** a School of Psychology, University of Nottingham, UK; b Nuffield Department of Clinical Neuroscience, University of Oxford, UK; c Department of Academic Radiology, University of Nottingham, UK; d Institute of Mental Health, School of Medicine, University of Nottingham, UK

**Keywords:** Diffusion tensor imaging, Tourette syndrome, Probabilistic tractography, Graph theory, Premonitory urges

## Abstract

Tourette syndrome (TS) is a neurodevelopmental disorder characterised by repetitive and intermittent motor and vocal tics. TS is thought to reflect fronto-striatal dysfunction and the aetiology of the disorder has been linked to widespread alterations in the functional and structural integrity of the brain. The aim of this study was to assess white matter (WM) abnormalities in a large sample of young patients with TS in comparison to a sample of matched typically developing control individuals (CS) using diffusion MRI. The study included 35 patients with TS (3 females; mean age: 14.0 ± 3.3) and 35 CS (3 females; mean age: 13.9 ± 3.3). Diffusion MRI data was analysed using tract-based spatial statistics (TBSS) and probabilistic tractography. Patients with TS demonstrated both marked and widespread decreases in axial diffusivity (AD) together with altered WM connectivity. Moreover, we showed that tic severity and the frequency of premonitory urges (PU) were associated with increased connectivity between primary motor cortex (M1) and the caudate nuclei, and increased information transfer between M1 and the insula, respectively. This is to our knowledge the first study to employ both TBSS and probabilistic tractography in a sample of young patients with TS. Our results contribute to the limited existing literature demonstrating altered connectivity in TS and confirm previous results suggesting in particular, that altered insular function contributes to increased frequency of PU.

## 1 Introduction

Hyperkinetic movement disorders, particularly those whose aetiology involve the basal ganglia (BG) nuclei and have a high rate of co-occurring neuropsychiatric disorders, have often been shown to exhibit alterations in brain white matter (WM) and grey matter (GM) (see Hayhow et al., 2013). One type of a hyperkinetic disorder, Tourette syndrome (TS), is of particular interest due its high rate of co-occurring neuropsychiatric disorders (Freeman et al., 2000, Zohar et al., 1999); complex aetiology (Du et al., 2010); its high impact on patients' social functioning and overall quality of life (Cath et al., 2011); and its multi-faceted symptoms which the patient can hide or mask for brief periods of time (Leckman, 2002). TS is characterised by rigid, repetitive, and intermittent movements and vocalisations termed tics (Leckman, 2002). TS is commonly diagnosed in early childhood, with the onset of tics reported to range between 5 and 10 years of age (Leckman, 2002). Tics tend to peak in severity around adolescence and may decrease in severity during early adulthood. Nonetheless, nearly a quarter of patients continue to present symptoms as adults and these may increase in severity and become resistant to treatment (Cath et al., 2011). Most frequently, patients present co-occurring symptoms of obsessive-compulsive disorder [OCD] and attention-deficit hyperactivity disorder [ADHD] (Freeman et al., 2000, Zohar et al., 1999). The prevalence of TS has been reported to be approximately 50 per 10.000 children, but rates are lower in adolescents and younger adults (Comings et al., 1990, Nomoto and Machiyama, 1990). The syndrome is known to be more prevalent in males than females with the gender ratio being ∼4:1 in child and adolescent samples (Tallur & Minns, 2010).

Patients with TS often also report the presence of uncomfortable bodily and cognitive urges referred to as sensory phenomena (SP). These SPs involve premonitory urge sensations (PU) which are thought to occur in almost all patients and are often localised to the area of tic (Cavanna et al., 2017, Kwak et al., 2003, Leckman et al., 1993). Frequency of PU in patients is usually measured using self-report questionnaires (Rosario et al., 2009, Woods et al., 2005). Patients often report that PU and tics coincide, and consequently, it has been proposed that tics are triggered by a conscious impulse, in which the tic soothes the uncomfortable sensation (SP) for a brief period of time (Bliss, 1980, Kane, 1994, Patrick, 1905). However, the causal link between tics and PU is currently unknown.

TS is widely characterised as a fronto-striatal disorder and the syndrome aetiology is thought to involve widespread alterations in the functional and structural integrity of the brain. Most commonly, symptoms of TS are attributed to disinhibition of cortico-striato-thalamo-cortical (CSTS) circuits (Albin and Mink, 2006, Mink, 2001). Mink (2001) proposed a model suggesting dysfunction of inhibitory control within the BG. This model linked the occurrence of tics to the altered activity of striatal neurons. Supporting evidence for this proposal was described in two neuropathological studies that demonstrated altered number and distribution of GABAergic and cholinergic interneurons within the striatum in severe and persistent TS (Kalanithi et al., 2005, Kataoka et al., 2010). Recently, animal and computational models have demonstrated that tic production can result from atypical selection processes employed by the CSTS circuits (Caligiore et al., 2017, McCairn et al., 2013).

Structural neuroimaging studies have shown reduced volume of several BG nuclei such as the caudate nucleus (Peterson, et al., 2003), thalamus (Makki et al., 2008, Makki et al., 2009) and regional increases in GM volume in the putamen (Ludolph et al., 2006) in children and adolescent patients with TS in comparison with typically developing control subjects. Paradoxically, a recent, multicentre study with a sample of young patients reported greater GM volume in the posterior thalamus but was unable to replicate previous morphological differences in the caudate (Greene et al., 2017). Also, thinning of sensorimotor cortices in TS correlating with severity of tic symptoms suggests a putative role of both subcortical and sensorimotor areas in the aetiology of TS (Sowell et al., 2008, Worbe et al., 2010). Several studies using diffusion tensor imaging (DTI) have shown altered properties of WM microstructure in patients with TS (e.g., Govindan et al., 2010, Jackson et al., 2011a, Jackson et al., 2011b, Makki et al., 2008). However, the majority of DTI studies have included adult patients with TS, while few studies have included children or adolescent patients. Therefore, there is great need to investigate WM alterations in young patients. This is particularly important as one study showed clear differences in structural integrity between adult and young patients with TS (Plessen et al., 2004). DTI is a powerful method of assessing structural integrity of WM in the brain non-invasively (Basser, Mattiello, & LeBihan, 1994). Most importantly, these studies have demonstrated abnormal diffusivity in subcortical structures (Govindan et al., 2010, Makki et al., 2008, Saporta et al., 2010), corticocortical association and fronto-striatal pathways, and insular cortices (Govindan et al., 2010). One recent study in very young patients demonstrated a widespread increase in axial diffusivity (AD; Liu et al., 2013) with another study showing a negative relationship between mean diffusivity and tic severity (Govindan et al., 2010). Similarly, altered water diffusion in the corpus callosum has been reported in several studies (Jackson et al., 2011a, Plessen et al., 2006, Wolff et al., 2016), although one study found no differences across groups (Jeppesen et al., 2014). Collectively, these results are consistent with the hypothesis of altered structural integrity of CSTC circuitry in TS, and potentially reduced functionality of information transfer across hemispheres that may contribute to symptoms through reduced inhibition of cortical activity (Boroojerdi et al., 1999, Orth and Rothwell, 2009). Unfortunately, there is little consensus across all of these studies since patient characteristics vary widely, e.g., comorbidities and medication status, rendering comparison difficult.

Using DTI tractography, two separate studies have demonstrated increased thickness and density of fibre bundles in prefrontal WM tracts (Xu et al., 2007) and decreased probability of connections between the left hemisphere caudate and prefrontal cortex (Makki et al., 2009). With DTI tractography it is possible to obtain estimates of brain network properties by applying graph theoretical analysis to the resulting connectivity matrices.

Graph theory is a nascent way of exploring both functional and structural integrity of the brain (Bullmore & Sporns, 2009). In graph theory, networks are characterised by vertices (nodes) and edges (links) indicating a connection between a pair of nodes (Bullmore & Sporns, 2009). To date, two resting-state fMRI studies have applied graph theory in the study of TS. Worbe et al. (2012) reported extensive alterations in CSTC networks in patients. These alterations were primarily a complete absence of hub-like nodes, an increase in short-range connections, and the overall increase in the number of connections. Furthermore, key nodes within networks that were assessed in that study correlated with tic severity and complexity. Lastly, Tinaz, Malone, Hallett, and Horovitz (2015) showed that patients demonstrate decreased connectivity in the parietal, limbic and, motor cortex regions, and increased connectivity of the thalamus and dorsal anterior insula in the right hemisphere.

Interestingly, only one study has explored the structural integrity of the brain using WM tractography and graph theory in young patients with TS (Wen et al., 2017). Also, no study has yet attempted to characterise the association between structural integrity of WM and measures of PU in patients with TS. This association been assessed using functional MRI. Bohlhalter et al. (2006) associated several areas of the brain including the insular cortex with urges experienced immediately prior to tics, suggesting a specific urge-to-tic network. Similarly, the GM volume of the dorsolateral putamen, the primary somatosensory cortex (Draganski et al., 2010), and the right insular cortex (Draper, Jackson, Morgan, & Jackson, 2015) have been associated with increased severity of PU, while resting-state functional connectivity between anterior insula and supplementary motor area has been shown to correlate with PU (Tinaz et al., 2015).

In summary, both structural and functional studies suggest altered volumes, impaired connectivity and abnormal water diffusion in key areas of the CSTC circuits in patients with TS. In this study, we used DTI to assess WM microstructure in a large sample of children and adolescents with TS and a matched group of typically developing controls using tract-based spatial statistics (TBSS) and probabilistic tractography. Two separate hypotheses are tested in this study. First, we want to establish that patients with TS demonstrate altered water diffusion in key WM pathways and, second, we hypothesise altered structural connectivity between cortical and subcortical regions of the brain characterised by loss of hub-like nodes in a network, greater number of connections and increased number of short range connections. Finally, we want to ascertain whether alterations in network attributes in patients with TS can be predicted using measures of tic severity and PU.

## 2 Materials and methods

### 2.1 Participants

The study was carried out in 35 children and adolescents (3 females, mean age: 14.0 ± 3.3, age range: 8.6–21.8) diagnosed with TS according to DSM-IV (APA, 2000) and 35 typically developing controls (CS; 3 females, mean age: 13.9 ± 3.3, age range: 8.5–21.2) matched for age and gender. Patients with TS were recruited from a specialist TS clinic based at the Queen's Medical Centre, Nottingham. Current symptoms of TS were assessed using the Yale Global Tic Severity Scale (YGTSS; Leckman et al., 1989). Participants were encouraged to describe tics specifically with regard to the frequency and intensity of their tics. Premonitory urges (PU) were assessed using the Premonitory Urge for Tics Scale (Woods et al., 2005). All participants were screened for symptoms of: ADHD using the Conners-3 self-report measure (Conners, 2008); obsessive-compulsive behaviour (OCB) using Children's Yale-Brown Obsessive Compulsive Scale (CY-BOCS; Scahill et al., 1997); and autism spectrum disorders (ASD) using Social Communication Questionnaire (SCQ; Berument, Rutter, Lord, Pickles, & Bailey, 1999) by a trained clinical research nurse. IQ estimates were obtained from each participant using the vocabulary and matrix reasoning sub-tests from the Wechsler's Abbreviated Scale of Intelligence (WASI; Wechsler, 1999). Exclusion criteria included an IQ below 70, diagnosis of anxiety, depression or any other form of psychiatric disorder, and learning and sight disabilities. Written informed consent was obtained from all patients (or their guardians). The study was approved by an appropriate local ethical review committee.

### 2.2 MRI parameters

Brain imaging data from all participants was acquired using a Philips 3T Achieva MRI scanner (Best, The Netherlands) equipped with a 32-channel SENSE head-coil at the Sir Peter Mansfield Imaging Centre (SPMIC), University of Nottingham. Participants were placed supine and head first into the scanner. Head motion was minimized by inserting foam pads between the participants' head and the head coil. Participants also wore headphones for hearing protection that provided additional control over head motion. Participants were instructed to lie as still as possible during and in between scans. Diffusion images were collected using an Echo-Planar Imaging sequence with 48 slices with 2 mm slice thickness and no inter-slice gap, TR/TE = 7655/55 msec, voxel size = 1 × 1 × 2 mm^3^, FOV = 224 × 224, matrix = 112 × 112, 32 gradient directions and a diffusion weighting (b-value) = 1000 sec/mm^2^. The first volume in the sequence for each subject has no diffusion weighting (b = 0) and is used to align the brain to the diffusion-weighted data during analysis. In addition, we acquired high-resolution T1-weighted anatomical images using a magnetization-prepared rapid gradient echo (MPRAGE) sequence with the following parameters: TR/TE = 8.41 msec/40 msec, FOV = 256 × 256, flip angle = 8°, 180 contiguous axial slices with 1 mm^3^ isotropic resolution.

### 2.3 MRI image processing

All DTI data was pre-processed using FSL's (v. 5.0.8) FDT toolkit (Behrens et al., 2003, Smith et al., 2004). Diffusion-weighted images were corrected for subject motion and Eddy currents using affine registration to the non-diffusion weighted images (B0 volume). Next, the brain was extracted from the skull and excess air removed using the Brain Extraction Tool (BET v. 2.1; Smith, 2002) with a fractional intensity threshold of .3. Diffusion tensors were fitted to each voxel of the brain using DTIFIT within FSL. The output images generated included: the three eigenvectors (V_1_, V_2_ and V_3_) and eigenvalues (λ_1_, λ_2_, λ_3_); FA; and an MD map at each brain voxel for each subject. The first eigenvalue (λ_1_) was used to represent AD and average of the second and third eigenvalues (λ_2_+λ_3_/2) to represent radial diffusivity. For each participant, we quantified head motion. Visual inspection was conducted for all data after every processing step to ensure data quality and to screen for any obvious MR scanning artefacts. Head motion parameters were calculated to provide a quantitative measure of movement inside the scanner. These were derived from the three translation (x, y, z in mm) and three rotation (α, β, γ, in radians) parameters. Rotational parameters were converted from radians to degrees at radius of 50 mm. Participants were excluded if any obvious distortions or artefacts were present during visual inspection or if any of the rotation or translation parameters exceeded 1.5 mm/degree.

### 2.4 TBSS

Diffusion maps were entered into a voxelwise statistical analysis using TBSS(Smith et al., 2006) to quantify differences in WM microstructure between patients and controls. TBSS projects volumetric data onto a WM skeleton. This method gains statistical power due to dimensionality reduction and thus circumvents the partial volume effect (Smith et al., 2006). TBSS is an attractive method due to its power in adjusting for image misalignment and renders data smoothing unnecessary. The TBSS processing pipeline consisted of nonlinear registration using the FLIRT function and b-spline representation of the registration warp field (Rueckert et al., 1999). Next, since the FMRIB58_FA target might be unsuitable for our young sample the most representative subject (one that requires the least amount of warping to match every other subject) across both groups was identified and all FA maps aligned to this target. Both target and individual FA maps were transformed into standard MNI space. A WM skeleton was created from the mean FA image to represent centres of all tracts with an intensity threshold of .2 (to exclude GM and CSF) and each subject's aligned FA image projected to the skeleton. Between-group comparison on FA maps was accomplished using this skeleton. Furthermore, original nonlinear registration of FA images can be used on other diffusion scalars (i.e., MD, AD and RD images), creating WM skeletons for each diffusion scalar (i.e., MD skeleton, RD skeleton, AD skeleton).

### 2.5 Region specific connectivity by probabilistic tractography

#### 2.5.1 Regions of interest

In addition to TBSS, region specific probabilistic streamline tractography was accomplished using the open-source diffusion MRI toolkit Camino (Cook et al., 2006), and using bayesian techniques (Friman, Farnebäck, & Westin, 2006) to track WM fibres between each region of interest (ROI) and to construct connectivity matrices for each participant in native space. With Bayesian techniques, the probability distribution function can be estimated on the principal fibre direction at each voxel in the brain and distribution of connectivity values between a particular seed and all other points are generated by repeatedly sampling connected pathways (Friman et al., 2006).

**Fig. 1:**
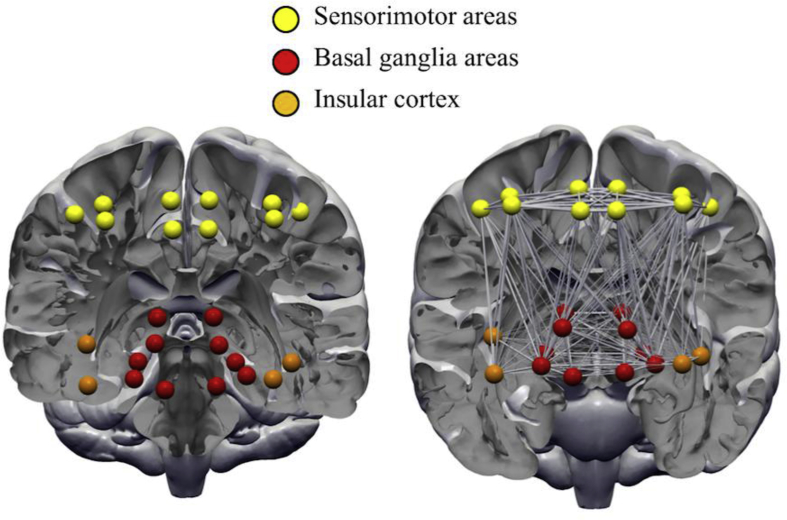
Regions of interests (ROIs) used in the probabilistic tractography analysis. ROIs were represented as nodes in a graph theoretical analysis. Yellow nodes = sensorimotor areas; red nodes = basal ganglia areas; orange = anterior and posterior insular cortex.

Twelve ROIs (Fig. 1) were carefully selected *a priori* ROIs based upon previous studies (Tinaz et al., 2015, Worbe et al., 2015) since we wanted to assess connectivity between cortical, subcortical and insular GM structures. ROIs were extracted from established and widely used templates available in the literature. Specifically, we used sensorimotor ROIs specified in the human motor area template (HMAT; Mayka, Corcos, Leurgans, & Vaillancourt, 2006), including the primary motor and sensory cortices (M1 and S1), SMA and pre-SMA, and the dorsal premotor cortex. Next, subcortical structures were selected based on the BG human area template (BGHAT; Prodoehl, Yu, Little, Abraham, & Vaillancourt, 2008), including the caudate, putamen, globus pallidus and thalamus, in addition to adding the nucleus accumbens (part of the ventral striatum). Finally, we included the anterior and posterior portions of the insular cortex, extracted from the comprehensive parcellation analysis done by Kelly et al. (2012) whose analysis was based on over 1000 MR scans derived from the 1000 Functional Connectomes Project.

Registration of ROIs from MNI space to individual DTI native space was accomplished in three steps: 1) Individual anatomical images were co-registered to the B0 image using linear transformation (FLIRT), 2) the transformed T1-weighted images were next transferred to a standard template [ICBM152 Montreal Neurological Institute (MNI)] space using linear and non-linear transformation (FLIRT and FNIRT), and 3) the resulting transformation matrix was applied inversely to bring each ROI into DTI native space (i.e., each ROI had a voxel dimension of 1 × 1 × 2 mm^3^). Distinct labelling of the ROIs was preserved using nearest neighbour interpolation. Finally, individual ROIs were combined into a single mask.

#### 2.5.2 Assessment of connectivity

Transformed, co-registered, T1-weighted images were segmented into GM, WM and CSF using the FAST segmentation tool in FSL (Zhang, Brady, & Smith, 2001). Whole-brain probabilistic streamline tractography was accomplished using the ‘track’ command within Camino. Segmented WM served as a starting seed for fibre tracking in a whole-brain technique termed ‘exhaustive search method’ (Basser et al., 2000, Gong et al., 2009, Mori and Van Zijl, 2002). Individual FA maps were entered to restrict the tracking procedure to voxels with FA ≥ .2 to exclude tracts entering GM or CSF. Tracking was initiated from the centre of each WM voxel with a step size of .5 mm. Tracking continued until a voxel was entered which fell below the termination criteria or when local streamline curvature exceeded 60°. Next, connectivity matrices were generated by recording how many streamlines connect each pair of ROI targets. Mean tract statistics from DTI scalars were extracted from each connected ROI pair. As a result, an undirected weighted structural brain network for each participant was represented by a symmetric 24 × 24 matrix. Finally, since raw streamline connectivity matrices might be influenced by brain volume we calculated total intracranial volume (TIV) as the sum of WM and GM.

#### 2.5.3 Network construction

We used graph theoretical analysis to quantify any differences in connectivity across the 24 ROIs. A graph is represented by *V* number of nodes connected by *E* edges (Bullmore & Sporns, 2009). An important step in graph theory analysis is assessing networks at different thresholds. However, few guidelines exist on the choice of these thresholds. Therefore, in this study networks were constructed based on a proportional thresholding method such that any connection value above an arbitrary percent threshold (i.e., .15 or 15%) were included as a connection, while other connections falling below this threshold were excluded. Subsequently, matrices were binarized and entered into the analysis. All analyses were conducted across a variety of thresholds ranging from .15 to .40 in steps of .01, equalling to 26 thresholds. Proportional thresholding ensures network compatibility across groups by keeping the number of nodes and edges the same and improves network measure stability (Garrison, Scheinost, Finn, Shen, & Todd, 2015). Nine graph metrics: global efficiency; small-worldness; assortativity; density; degree; betweenness centrality; shortest path length; clustering coefficient; and local efficiency, were calculated and are detailed in Table 1. It is possible that with 24 ROIs our networks could lack small-world properties, which is an essential factor in graph theory. According to the seminal work done by Watts and Strogatz (1998) and further iterated by others (e.g., Humphries, Gurney, & Prescott, 2006) small worldness can be confirmed in networks using real and random network clustering coefficient and characteristic path length. Therefore, to examine the small worldness of our networks we calculated the normalised clustering coefficient (γ) and normalised path length (λ). The small-world coefficient σ is then calculated where σ = γ/λ. If σ > 1, a network can be considered to reflect small-world properties. Finally, we defined ‘hubs’ using the following criteria: betweenness centrality and degree were normalised across nodes (z-score) and thresholded at z ≥ 2. Hubs are thought to promote integration between different parts of functional networks.

**Table 1:** Graph metrics used in the study.


Assortativity	A correlation coefficient between degrees of all nodes between pair of nodes. When assortativity is positive it indicates that nodes tend to connect to other nodes with same or similar degree.
Betweenness centrality	High values indicate that nodes participate in a greater number of shortest paths.
Characteristic path length (local)	Average shortest path length of a node to the rest of the network.
Clustering coefficient	The fraction of a nodes neighbours which are also neighbours of each other. This is zero if nodes are isolated or only connect with one other node.
Degree	Total number of links connected to a node.
Density	A fraction of present connections to all possible connections in a network.
Network global efficiency	Average inverse shortest path length in the network.
Network local efficiency	This is calculated as global efficiency on node neighbourhoods.
Small-worldness	Measures the extent of how tightly clustered the network is.

### 2.6 Statistical analysis

Statistical differences in demographics between the two groups and head motion parameters were determined using parametric *t*-tests.

#### 2.6.1 TBSS

When assessing differences in water diffusivity, age and IQ were de-meaned and entered as variables of no interest to remove any confounding differences between the two groups (TS *vs*. CS). Diffusion maps were entered into voxelwise cross-subject statistics in FSL with 5000 permutations using the inbuilt function ‘randomise’ (Winkler, Ridgway, Webster, Smith, & Nichols, 2014). For all analyses, multiple voxel-wise comparison was controlled using family-wise error correction with a threshold set at *p* < .05 and cluster size of ≥20 voxels. Therefore, all TFCE value images reported here were fully corrected for multiple comparisons. In addition, we ran correlation analyses in patients with TS only to assess whether any TBSS values correlated significantly with PU (PUTS) and/or tic severity scores. Clinical scores and patient diffusion maps were entered into a linear regression model in FSL with age and IQ as nuisance variables.

Tracts were identified using the JHU White-Matter Tractography and JHU ICBM-DTI-81 White-Matter Label atlases embedded within the FSL package.

#### 2.6.2 Graph metrics

Graph metrics were assessed across groups using a general linear model (GLM) with group as a between subjects factor and age, IQ and TIV as nuisance covariates. We generated 500 null-networks, with the same number of nodes and edges, and degree distribution as the real networks for each subject (Maslov & Sneppen, 2002). These null networks can be used for normalisation of network measures such as small-worldness (i.e., to produce the normalised γ, λ, and σ metrics). We then used a multiple linear regression to assess any association between graph metrics and clinical measures in patients only. As such, we ran the association analysis using the two clinical measures separately, between all nodes and across all thresholds and all graph metrics. Thus, 26 × 24 × 1 × 9 = 5,616 correlation coefficients were corrected for using multiple comparison correction. We also note that we ran the association analysis in patients only since the two clinical measures (YGTSS and PUTS) were not recorded in the control group.

Statistical differences in graph metrics were assessed using 1000 permutation tests. Statistical difference was first explored at a more lenient *p* < .001 uncorrected threshold. *p*-values were corrected for multiple comparison using false discovery rate (FDR; Benjamini and Hochberg, 1995) at p-FDR<.05. Graph metrics and statistical analysis was accomplished using the Graphvar interface for the Brain Connectivity Toolbox (Kruschwitz, et al., 2015; Rubinov & Sporns, 2010).

## 3 Results

### 3.1 Sample characteristics

Post pre-processing showed that 7 patients with TS and 5 control subjects had to be excluded due to excessive head motion during the DTI scan as measured using rotation and translation parameters. The final sample therefore consisted of 28 patients with TS (3 females, mean age: 14.5 ± 3.8, age range: 8.6–21.8) and 30 control subjects (2 females, mean age: 14.1 ± 2.9, age range: 9.6–21.2). Details of the patient sample can be seen in Table 2. In our sample, eight patients were medicated at the time of scanning. Nine out of 28 patients had no comorbidities, six had comorbid ADHD, two had OCD, and eight had combined ADHD, OCD and/or ASD. One participant was not screened. All patients reported PU. IQ was not significantly different between the two groups in our final sample (TS mean: 110.1 ± 14.6, CS mean: 119.9 ± 12.1, *p* > .05).

**Table 2:** Patient specific characteristics.

TS ID	Sex	Age (years)	Yale Motor tic score	Yale Phonic tic score	Yale Impairment score	YGSS	Motor tics		Phonic tics		PUTS	Comorbidity	Medication
							Simple	Complex	Simple	Complex			
TS01	M	19.3	18/25	9/25	5/50	32/100	+	+	+	–	18/36	None	None
TS02	M	17.0	12/25	11/25	0/50	23/100	+	+	+	+	10/36	None	None
TS03	M	20.1	21/25	16/25	30/50	67/100	+	+	+	+	22/36	OCD, ADHD, possible ASD	None
TS04	M	15.4	3/25	0/25	0/50	3/100	+	–	–	–	14/36	None	None
TS05	M	15.0	13/25	15/25	5/50	32/100	+	+	+	+	18/36	OCD, ADHD	Sertraline and Melatonin
TS06	M	15.6	13/25	11/25	10/50	34/100	+	+	+	+	27/36	None	Clonidine
TS07	M	11	12/25	9/25	5/50	26/100	+	+	+	+	23/36	ADHD	None
TS08	M	15.1	21/25	17/25	25/50	63/100	+	+	+	+	28/36	OCD, ADHD	Melatonin
TS09	M	14.5	7/25	4/25	0/50	11/100	+	–	+	–	14/36	ADHD	None
TS10	M	15.6	10/25	11/25	10/50	31/100	+	–	+	+	21/36	ADHD	None
TS11	M	16.2	12/25	8/25	10/50	30/100	+	–	+	–	17/36	OCD	Aripiprazole 5 mg
TS12	F	12.6	14/25	8/25	20/50	45/100	+	–	+	–	20/36	OCD	Melatonin
TS13	F	21.8	17/25	10/25	5/50	32/100	+	+	+	–	20/36	ASD	Citalopram 40 mg
TS14	M	12.8	15/25	10/25	5/50	30/100	+	+	+	–	23/36	None	None
TS15	M	10.1	12/25	8/25	5/50	25/100	+	+	+	–	17/36	None	None
TS16	M	15	12/25	14/25	5/50	31/100	+	+	+	+	21/36	ADHD, ASD	None
TS17	F	18.7	12/25	15/25	5/50	32/100	+	+	+	+	21/36	ADHD, ASD, possible OCD	None
TS18	M	14.5	13/25	0/25	10/50	23/100	+	–	–	–	33/36	ADHD	Clonidine 50 mcgs, Aripiprazole 5 mgs
TS19	M	8.6	10/25	3/25	10/50	23/100	+	+	+	–	18/36	None	None
TS20	M	15.3	10/25	13/25	10/50	33/100	+	+	+	+	30/36	Mild OCD, ADHD	None
TS21	M	10.5	16/25	14/25	10/50	40/100	+	+	+	+	20/36	Mild OCD, ADHD	None
TS22	M	14.1	10/25	9/25	5/50	24/100	+	–	+	–	18/36	ADHD	None
TS23	M	18.9	22/25	22/25	22/50	64/100	+	+	+	+	17/36	None	None
TS24	M	10.3	13/25	7/25	5/50	25/100	+	–	+	–	15/36	None	None
TS25	M	12.2	3/25	3/25	0/50	6/100	+	–	+	–	10/36	ADHD	None
TS26	M	16.4	23/25	15/25	40/50	78/100	+	+	+	+	23/36	Unknown	Aripiprazole 10 mg, Sertraline 100 mg and Clonidine 200 mg
TS27	M	8.7	13/25	11/25	5/50	29/100	+	+	+	–	21/36	ADHD	None
TS28	M	12.4	15/25	15/25	30/50	60/100	+	+	+	+	32/36	Mild OCD, ADHD	None
*N* = *28*		*14.5 ± 3.4*	*13.2 ± 4.7*	*10.3 ± 5.2*	*10.4 ± 10.2*	*33 ± 15.3*					*20.4 ± 5.8*	–	–

Abbreviations: YGSS: Yale Global Severity Scale; PUTS: Premonitory urge for tics scale; OCD = Obsessive compulsive disorder; ADHD = Attention-deficit hyperactivity disorder; ASD = Autism spectrum disorder; + = symptom is present; – = symptom is not present.

### 3.2 Between-group differences in voxel-wise cross-subject statistics

**Fig. 2:**
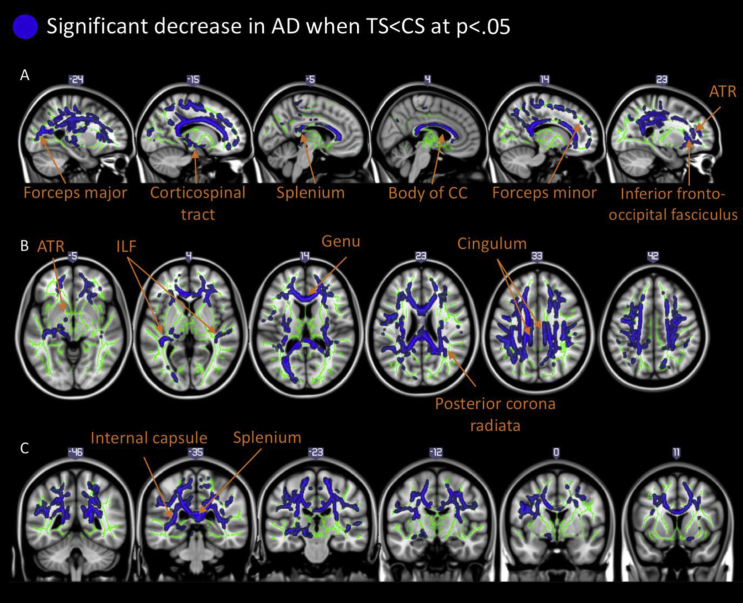
Illustrates key tracts exhibiting significant decreases in AD (*p* < .05 corrected for multiple comparison). Differences are projected onto the mean WM skeleton (green) and the MNI 152 template. Top panel, A: sagittal images showing significant tracts. Coordinate numbers are X coordinates in MNI space. Middle panel, B: axial images showing significant tracts. Coordinate numbers displayed are Z coordinates in MNI space. Bottom panel, C: images showing significant tracts. Coordinate numbers are Y coordinates in MNI space. Abbreviations, ATR = anterior thalamic radiation. CC = corpus callosum. ILF = inferior longitudinal fasciculus.

Results of the comparison between the group of patients with TS and matched control subjects demonstrated that there were widespread decreases in AD values in the TS group bilaterally (one large cluster of 32,988 voxels). Relevant data are presented in Fig. 2. Four peak coordinates were identified being the right anterior thalamic radiation [ATR; MNI coordinates: X: 23 Y: −40 Z: 28, t (56) = 2.99, *p* < .05], the splenium of the corpus callosum [MNI coordinates: X: −11 Y: −35 Z: 24, t (56) = 3.00, *p* < .05], the genu of the corpus callosum [MNI coordinates: X: −11 Y: 20 Z: 20, t (56) = 2.56, *p* < .05] and the left forceps minor [MNI coordinates: X: −12 Y: 36 Z: 4, t (56) = 2.70, *p* < .05]. We did not find any significant increases of AD in patients compared to controls. Furthermore, there were no differences between the two groups for FA or RD at *p* < .05. Finally, we found no effect of medication since a separate TBSS analysis, excluding patients on medication, yielded the same results demonstrating widespread decrease in AD in TS, but unaffected FA and RD.

### 3.3 Relationship between water diffusion and clinical measures in patients with TS

We found no significant association between any diffusion measure (FA, AD, MD or RD) and PU (PUTS) or tic severity (Yale score) in patients (all *p* > .05).

### 3.4 A trend for altered WM connectivity in patients

Analysis of tractography data was carried out using AD maps for each subject. This was preferred over using the more conventional FA maps since our TBSS result showed significant group differences on AD maps. We are aware that AD is perhaps not as representative as a connectivity measure in comparison to FA. However, FA is a composite measure of all diffusion directions, while AD represents the diffusion parallel to the principal axis of the tensor. We also believe that using AD, which is clearly altered in TS (whereas FA is not), adds depth to the discussion of the pathology of TS. Therefore, for each pair of ROIs we extracted the mean AD value of WM tract that connects the two.

**Fig. 3:**
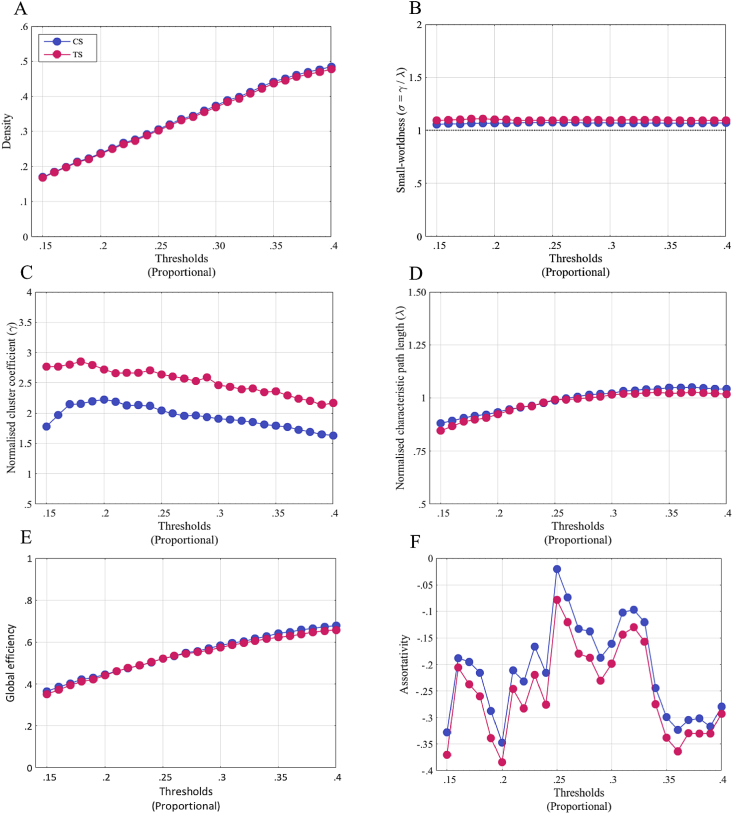
Comparison of global metrics between groups. Global metrics: A) density, B) small-worldness, C) global efficiency and D) assortativity, were compared across the two groups. None of the global metrics were significantly different across groups (all *p* > .05).

**Fig. 4:**
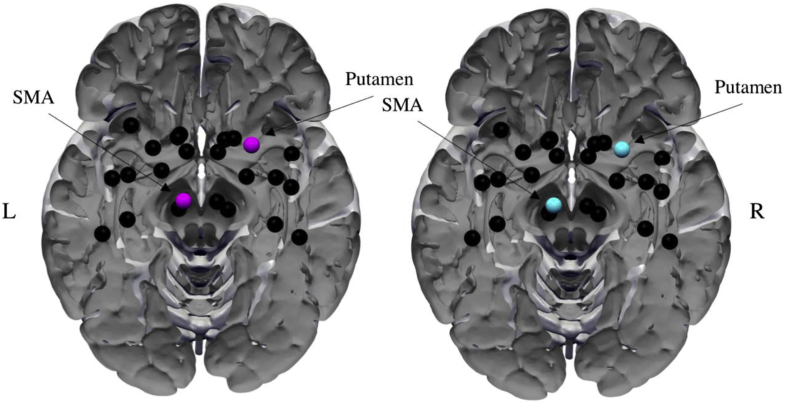
Hubs. Nodes with hub-like characteristics were constructed from betweenness centrality and degree metrics. Nodes with z-score ≥2 across both metrics were considered to show hub-like characteristics. Two nodes, left SMA and right Putamen showed such characteristics in both groups.

Network density was not significantly different across groups at any threshold [Fig. 3A; t (56) = .61–.68, *p* > .001]. This indicates that the any observed differences between groups in graph metrics cannot be attributed to differences in network density. No other global metric (i.e., small-worldness, γ, λ, global efficiency, and assortativity) showed any significant difference across groups (Fig. 3B–F; all *p* > .05). By contrast, we found significantly enhanced betweenness centrality with respect to the left putamen of the TS patients at several thresholds [thresholds: 25-24%, 22-19%, CS grand mean: 39.6, TS grand mean: 46.65, t (56) = −1.69 to −2.17, *p* < .001 uncorrected]. The difference in betweenness centrality became non-significant after stringent multiple comparison correction, and results are interpreted with this in mind. The characteristic path length was also significantly higher in patients across several thresholds for the right accumbens [thresholds: 28%–20%, CS grand mean: 4.30, TS grand mean: 4.62, t (56) = −1.37 to −1.89, *p* < .001 uncorrected]. No threshold survived our stringent multiple comparison correction. No other graph metric showed any significant between group differences. Additionally, we calculated hubs from metrics betweenness centrality and degree. Nodes satisfying the condition of having z-scores ≥2 across both metrics were considered as hubs. As can be seen in Fig. 4, both groups had two nodes showing hub-like characteristics, the right putamen and left SMA.

### 3.5 Association between graph metrics and clinical measures

**Fig. 5:**
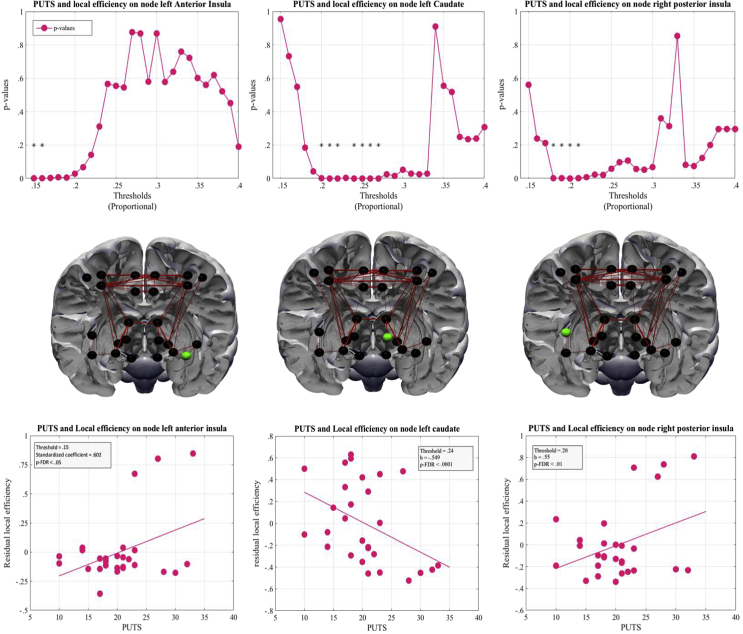
Local metrics showing association with PUTS scores. Local efficiency of three nodes showed a significant association with PUTS scores. Column A shows results from the left anterior insula; Column B shows results from the left caudate; Column C shows results from the posterior insula. Top row: *p*-values across thresholds. Middle row; nodes are coded in green. Bottom row: regression plots showing relationship between local efficiency and PUTS scores. *p-FDR<.05.

We used multiple linear regression analysis to investigate any association between the different graph metrics (adjusted for the effects of age, IQ and TIV) and tic severity (YGTSS) or premonitory urges (PUTS) in TS patients. PUTS scores showed a significant association with local efficiency on three nodes (ROIs). First, the left caudate was negatively associated with PUTS scores [thresholds: 27%–24%, 22-20%, β = −.55 to −.57, t (22) = −2.66 to −2.94, *p* < .001]. Second, there was also a significant positive association with PUTS scores for the right posterior insula in four threshold [thresholds: 21-18%, β = .55, t (22) = 2.79, *p* < .001]. Third, there was a positive association with PUTS scores for the left anterior insula [thresholds: 16-15%, β = .60, t (22) = 3.14–3.25, *p* < .001]. All thresholds survived correction for multiple comparisons. The relationship between PUTS scores and the residual local efficiency is depicted in Fig. 5. Tic severity scores and measures of betweenness centrality showed a significant positive association on three nodes. The left thalamus [thresholds: 28%–15%, β = 6.66–17.35, t (22) = 1.82–3.79, *p* < .001] with two thresholds surviving FDR correction; the right M1 [thresholds: 40-38%, 35–3% and 29–15%, β = 7.59–16.27, t (22) = 1.51–3.49, *p* < .001], with one threshold surviving FDR correction; and the right dorsal premotor cortex [thresholds: 40–33%, 20% and 18–15%, β = 7.97–16.42, t (22) = 1.44–3.45, *p* < .001] with no threshold surviving a multiple comparison correction. No association was found between clinical measures and any global metric (all *p* > .001). For brevity we decided not to describe or report any non-significant correlations (i.e., where *p* > .001 uncorrected).

## 4 Discussion

In this study, we used TBSS and probabilistic tractography to investigate alterations in WM microstructure in a large sample of children and adolescents with TS compared to a group of age- and gender matched typically developing controls. Our TBSS analysis revealed a marked and widespread *decrease* in AD in patients with TS (see Fig. 2.). In addition, patients also demonstrated altered WM connectivity measured using WM tractography. These results are in line with past studies showing altered WM microstructure in patients with TS (Govindan et al., 2010) and altered connectivity of fronto-striatal areas (Worbe et al., 2012, Worbe et al., 2015). However, we failed to note any significant alterations in the most commonly used diffusion metric (FA). Nonetheless, the novelty of our results is showing, for the first time in a sample of patients with TS, a direct relationship between structural connectivity and frequency of PU. This is, to our knowledge, the first study to employ both TBSS and probabilistic tractography to examine potential atypical WM microstructure in a young sample of patients with TS.

### 4.1 TS is associated with marked and widespread alterations in AD

DTI is used to describe diffusion of water in tissue and is useful since random motion of water molecules in the brain are influenced by architectural properties of tissue (Le Bihan, 2003). Diffusion of water in tissue varies with direction and is least restricted parallel to the orientation of the fibres, while being much more restricted perpendicular to them (Nucifora, Verma, Lee, & Melhem, 2007). Our results of marked and widespread *decrease* in AD is in contrast to recently published results by Liu et al. (2013), showing regional *increases* of AD in very early TS. However, this apparent discrepancy could be explained by differences in the age of participants. Our sample largely consisted of adolescents whereas participants in Liu et al.'s study were very young children with short disease duration. Age has previously been shown to be a significant factor in diffusion MR studies (Asato et al., 2010, Bennett et al., 2010). Specifically, we tend to see a significant linear decline in AD with age in healthy participants in almost all parts of the brain (Kumar, Nguyen, Macey, Woo, & Harper, 2012). Therefore, the two studies indicate that whereas AD is enhanced in very young patients this reverses by early adolescence, in comparison with healthy control subjects. It is difficult to interpret these findings as TS is not known to affect WM directly. However, we theorise that these differences seen in patients with TS are due to increased freedom of cross-fibre diffusion as the patient grows older, affecting AD (Alexander et al., 2007, Song et al., 2002). Therefore, the apparent discrepancy of our results and the ones by Liu et al.'s could also indicate a more rapid decline of AD values in patients with TS by increased branching of WM tracts. At present this is a postulation at best and a longitudinal study would clarify this. It is important to reiterate that our results cannot be attributed to differences in age of participants since age was used as variable of no interest. This is imperative since a recent study by Worbe et al. (2015) showed that diffusion was significantly influenced by age of participants.

Our sample of patients with TS also presented with several comorbid disorders such as ADHD and OCD (see Table 1). Interestingly, our TBSS results do conform to diffusion studies in patients with OCD but not ADHD. Young patients with ADHD are thought present with *increased* AD in several fronto-striatal, prefrontal and occipital WM pathways in comparison with control subjects (T. J. Silk et al., 2009, Tamm et al., 2012). However, AD seems to be significantly increased (there was no effect of any other diffusion scalar) in healthy controls in comparison with young OCD patients in both the genu and splenium of the CC (T. Silk, Chen, Seal, & Vance, 2013). These results indicate that whereas AD seems to be increased in ADHD patients, AD seems to be diminished in TS and OCD patients. Unfortunately, we were unable to carry out any sub-group analyses due to lack of power. Plessen et al. (2006) showed however that there is a significant reduction of WM connectivity in the CC in patients with TS, and these results remained following subgroup analyses (TS + ADHD, TS + OCD).

We were unable to find any significant associations between clinical measures and measures of WM diffusion. This is in line with recent study by Jeppesen et al. (2014). By contrast, other studies have reported significant correlations. For example, Liu et al. (2013) showed that WM microstructure in the right ATR pathway and right cingulum were positively correlated with tic severity. Likewise, in a similar sample as in the current study we previously demonstrated that WM water diffusion in the CC and forceps minor positively predicted tic severity (Jackson, Parkinson, Jung, et al., 2011). These results were interpreted by Jackson and colleagues to indicate that having TS might induce a compensatory functional reorganization. Regression analyses in the current study suggest that syndrome related decreases in AD are not associated with tic severity or frequency of PU. Instead, structural differences in the brain can only be attributed to having TS.

### 4.2 A trend for altered structural connectivity in TS

In addition to TBSS, we also assessed WM connectivity using probabilistic tractography and graph theoretical analysis with a set of *a priori* specified ROIs to quantify any differences in structural connectivity across the two groups. ROIs were specified from sensorimotor areas, subcortical regions and the insular cortex (see Fig. 1). Tractography, in conjunction with high resolution anatomical images, gains information about inter-voxel connectivity and WM structural connectivity, non-invasively (Behrens, Berg, Jbabdi, Rushworth, & Woolrich, 2007). We used graph theory with our tractography data and found some support for our second hypothesis. Patients in our sample displayed an increased number of connections amongst our ROIs but also exhibited a less functionally integrated network, as measured using the characteristic path length metric. However, as our results did not survive a stringent multiple comparison correction, possibly due to lack of power, interpretation of these effects should be made with caution. Previously Worbe et al. (2012) reported a complete lack of hubs, more functionally integrated networks and increased number of connections in adult patients with TS. In our sample we found no hub-related differences between the two groups. Hubs are considered to be important nodes in a network, with a maximum number of connections, they also facilitate integration between parts of a network (Rubinov & Sporns, 2010). In our data, we identified two nodes (left SMA and right putamen) classified as hubs in both groups. Interestingly, this was also demonstrated in a recent paper where both patients and control subjects had highly similar hub distribution (Wen et al., 2017). Similarly, in adults, both the left SMA and right putamen have been shown to display increased betweenness centrality in patients (Tinaz et al., 2015). This indicates that amongst both children and adults, these two nodes might play a significant role in the pathology of TS.

### 4.3 Node specificity for tic severity and PU

To ascertain any relationship between clinical variables and graph metrics we ran a multiple regression analysis in our sample of patients. Most notably, we observed a positive linear association between the frequency of PU in patients and the efficacy of information transfer in the right posterior insula and left anterior insula as well as a negative relationship with information transfer in the left caudate after correcting for age, IQ and TIV. Thus, PUTS scores explain a significant amount of variance in local efficiency after the variance associated with these other variables has been controlled for. The insular cortex has been associated with interoception and activates significantly in fMRI studies during somatosensory stimulation and suppression of bodily urges (Berman et al., 2012, Jackson et al., 2011b). More specifically, the right posterior insula has been shown to be specific for action and perception while the left anterior insula is important for cognition and emotion (Kelly, et al., 2012). In the context of TS, it has been demonstrated that functional connectivity of the right dorsal anterior insula has a significant relationship with urge frequency and tic severity in adult patients with TS (Tinaz et al., 2015). Similarly, in children it has been shown that GM thickness of the left insula is negatively associated with PUTS scores (Draper et al., 2015).

We also noted that tic severity was significantly associated with betweenness centrality on two nodes; a positive correlation with the left thalamus and right M1, indicating that as tic severity increases in patients these two nodes participate in a greater number of shorter paths within the network and might act as aberrant hub-like nodes.

### 4.4 Implication for clinical practice

Although not the main focus of our study, it is interesting to consider how our results could be implemented into clinical practice. For example, our results of increased information transfer (i.e., efficiency of certain areas of the insula) contributing to increased frequency of PU suggests hyper-connectivity of the insula to the rest of the nodes in a network. It is yet to be explored whether this increased connectivity can be reversed with behavioural therapy. One previous study showed that comprehensive behavioural intervention for tics (or CBIT) resulted in decreased task-related activation of the putamen in patients with TS (Deckersbach et al., 2014). By contrast, typically developing individuals demonstrated increased activity in the same region. The authors interpreted this finding as to indicate that CBIT might reduce hyper-elevated activity of the putamen and improve inappropriate activation of the putamen. The efficacy of other non-pharmacological remedies such as high aerobic exercise has been shown to reduce the frequency of tics, outlasting the duration of the exercise (Nixon, Glazebrook, Hollis, & Jackson, 2014). No study has investigated the effect of behaviour therapy on WM connectivity. Nonetheless, the evidence exists that greater habitual behaviour in patients with TS is related to stronger WM connectivity within the cortico-striatal network. We therefore urge future studies to look at the effect of behavioural therapy or exercise on the integrity of WM pathways.

## 5 Conclusion

In this study we have demonstrated, for the first time in a young sample of patients with TS, clear atypical WM microstructure and evidence for altered structural connectivity using diffusion MRI in comparison to matched typically developing control subjects. These results add to the existing literature of altered connectivity of the insula which might play a role in the frequency of PU experienced by patients. We also show that in contrast to very young patients, AD decreases exponentially as the patient grows older.
